# Delayed Diagnosis of Pediatric Sternoclavicular Joint Infections and Clavicular Osteomyelitis During the COVID-19 Pandemic: A Report of 3 Cases

**DOI:** 10.5435/JAAOSGlobal-D-21-00302

**Published:** 2022-09-27

**Authors:** Elizabeth M. Benson, Ezan A. Kothari, Timothy W. Torrez, Michael J. Conklin, Stephanie Berger, Kevin A. Williams

**Affiliations:** From the Department of Orthopaedic Surgery, the University of Alabama at Birmingham, Birmingham, AL.

## Abstract

Sternoclavicular joint infections and osteomyelitis of the clavicle are extremely rare infections, especially in the pediatric population. Early signs of these infections are nonspecific and can be mistaken for common upper respiratory infections such as COVID-19 and influenza. Rapid diagnosis and treatment are critical for preventing potentially fatal complications such as mediastinitis. We present three cases of sternoclavicular joint infections in the past year during the COVID-19 pandemic. All three patients had delayed diagnoses likely secondary to COVID-19 workup. Each patient underwent surgical irrigation and débridement. Two of three patients required multiple surgeries and prolonged antibiotic courses. Placement of antibiotic-impregnated calcium sulfate beads into the surgical site cleared the infection in all cases where they were used. All three patients made a full recovery; however, the severity of their situations should not be overlooked. Children presenting to the hospital with chest pain, fever, and shortness of breath should not simply be discharged based on a negative COVID-19 test or other viral assays. A higher index of suspicion for bacterial infections such as clavicular osteomyelitis is important. Close attention must be placed on the physical examination to locate potential areas of concentrated pain, erythema, or swelling to prompt advanced imaging if necessary.

Sternoclavicular joint infections (SCJIs) and clavicular osteomyelitis are rare in the pediatric population and account for less than 1% of pediatric joint infections.^1,2^ Definitive diagnosis is with sternoclavicular joint (SCJ) aspiration or bone culture. Severe cases and cases refractory to antibiotics often require surgical irrigation and débridement (I&D).^3^

The most common presentation of SCJI is nonspecific chest pain, which can be mistaken for a cardiac or respiratory etiology.^4^ Early diagnosis and treatment of SCJIs is very important because of their proximity to vital structures of the mediastinum. Complications from SCJI can include mediastinitis, recurrent osteomyelitis, chest wall abscess, and death.^5,6,7,8^ It is critical to have a high index of suspicion for SCJI in a patient presenting with new-onset shoulder or chest pain even in the absence of overt signs of infection.

In this case report, we present three pediatric cases of SCJI, an exceedingly rare condition with only a handful of studies pertaining to children.^9^ All three cases share the common theme of delayed diagnosis, and all three patients presented during the COVID-19 pandemic.

COVID-19 has had a notable effect on the diagnosis and treatment of serious non–COVID-19 conditions such as acute myocardial infarction and acute appendicitis.^10,11^ The combination of the pandemic with this elusive condition creates a precarious situation. This report is intended to share our approach for the surgical management of SCJI in pediatric patients and to demonstrate the potentially dangerous effect that the COVID-19 pandemic has on its prompt recognition and treatment.

## Case Report

### Patient 1

Patient 1 is a 14-year-old adolescent boy who presented with dyspnea and left-sided neck and chest pain. He was seen at three outside facilities that diagnosed his condition as a pulled muscle and subsequently as cellulitis, which was treated with amoxicillin/clavulanate. The patient was negative for strep throat, infectious mononucleosis, COVID-19, and influenza.

**Figure 1:**
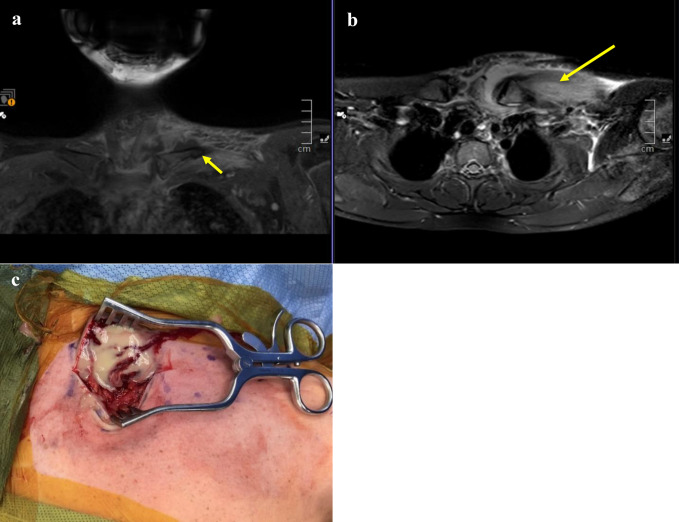
Patient 1 imaging. Preoperative coronal T1 postcontrast (**A**) and T2 axial (**B**) images revealing evidence of left SCJI with large effusion of SCJ, subperiosteal fluid without contrast enhancement (short arrow), chest wall edema (long arrow), and intramedullary edema, suggesting early osteomyelitis. **C**, Intraoperative image demonstrating purulence from the left sternoclavicular joint. SCJ = sternoclavicular joint, SCJI = sternoclavicular joint infection

His symptoms were present for 5 days on arrival. On physical examination, he had mild erythema of the left anterior chest wall, with a palpable nodule adjacent to the clavicle. There was limited range of motion of the left shoulder secondary to anterior chest wall pain. His laboratory results were significant for elevated erythrocyte sedimentation rate (ESR) (31 mm/h), c‐reactive protein (CRP) (6.15 mg/dL), and fibrinogen (549 mg/dL) (Table 1). He was started on intravenous (IV) vancomycin and ceftriaxone. CT imaging was concerning for cellulitis of the anterosuperior left chest wall, and MRI was significant for diffuse cellulitis, subperiosteal effusion, and possible SCJ septic arthritis (Figure 1, A and B).

**Table 1:** Patient Demographics and Clinical Summary

Patient	Age	Sex	Presenting Symptoms	Previously Diagnosed As	Physical Examination	Laboratory test results	Imaging	Treatment
1	14	M	Neck pain, chest pain, and SOB	Pulled muscle and cellulitis	Erythema and edema of the clavicle and chest wall. Palpable nodule on the clavicle. Limited shoulder ROM	WBC—8 PLT—196 ESR—31 CRP—6	Subperiosteal fluid collection and chest wall edema.	I&D and clindamycin (PO)
2	12	F	Fever and chest pain	Strep pharyngitis	Erythema and edema overlying the clavicle and SCJ. Tenderness to palpation of the clavicle, chest, and shoulder	WBC—10.5 PLT—290 ESR—69 CRP—14	Osteomyelitis clavicle, subperiosteal abscess, and SCJ septic arthritis	I&D (×3) and clindamycin (IV and PO)
3	14	M	Malaise, fever, and shoulder pain	Viral illness	Erythema, edema, and tenderness to palpation overlying the clavicle, sternum, and base of the neck. Limited shoulder ROM to flexion and abduction.	WBC—21 PLT—304 ESR—86 CRP—33	Osteomyelitis of the clavicle, subperiosteal abscess, SCM abscess, chest wall edema, and possible mediastinitis	I&D (×2), cefazolin (IV), and cephalexin (PO)

I&D = irrigation and débridement, IV = intravenous, PLT = platelet count, ROM = range of motion, SCJ = sternoclavicular joint infection, SCM = sternocleidomastoid, SOB = shortness of breath, WBC = white blood cell.

Normal CRP range: 0.00 to 0.50 mg/dL

**Figure 2:**
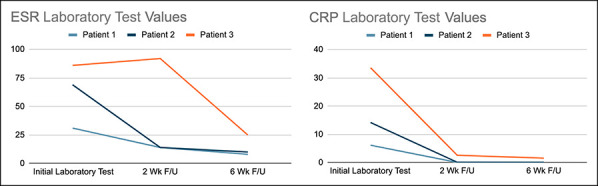
Graphs showing trended ESR and CRP values for patients 1 to 3. Follow-up is based on the time after last I&D. I&D = irrigation and débridement

Owing to imaging consistent with subperiosteal abscess and spread of infection to the adjacent joint, irrigation and débridement with fenestration of the clavicle was done (Figure 1C). Bone cultures were positive for methicillin-resistant *Staphylococcus aureus* (MRSA) with clindamycin susceptibility. He was transitioned to oral clindamycin for 6 weeks. Laboratory test results normalized by the 2-week follow-up (Figure 2). The patient was asymptomatic without evidence of recurrence after discontinuation of antibiotics.

### Patient 2

Patient 2 is a 12-year-old adolescent girl who presented with left-sided clavicular pain and fevers for 7 days. She was seen at two outside facilities where she tested negative for COVID-19. Owing to worsening symptoms, she was retested for COVID-19, which returned negative, but a rapid strep test was positive. She was prescribed amoxicillin for presumed strep pharyngitis, but her symptoms persisted.

**Figure 3:**
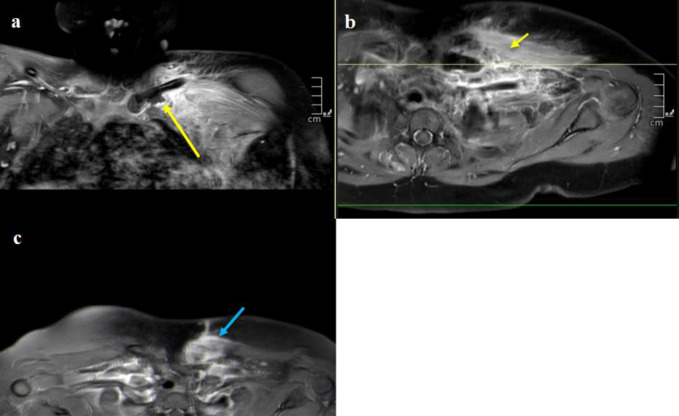
Patient 2 preoperative MR imaging. Coronal (**A**) and axial (**B**) T1 images after gadolinium administration demonstrating subperiosteal fluid collection (long arrow), likely due to metaphyseal extension from the epiphysis with surrounding edema in the chest wall musculature (short arrow), representing left SCJI. **C,** Subsequent T1 axial MR image with contrast 3 months after initial I&D demonstrating left medial clavicular osteomyelitis (blue arrow). I&D = irrigation and débridement, SCJI = sternoclavicular joint infection

Examination of the left shoulder revealed limited range of motion; erythema; edema overlying the SCJ; and exquisite tenderness to palpation of the proximal clavicle, upper chest, and shoulder. ESR and CRP were elevated, 69 and 14, respectively (Figure 2). The patient's white blood cell count was 10.5 and platelet count was 290,000 (Table 1). Radiographs were normal. MRI findings were consistent with acute clavicular osteomyelitis, subperiosteal abscess, and potential SCJ septic arthritis (Figure 3, A and B). Given the MRI findings, I&D with intramedullary drilling was done. Intraoperative cultures grew MRSA with clindamycin susceptibility. She was initially started on IV clindamycin and transitioned to per os (by mouth) (PO).

Twenty days postoperatively, the patient presented with malaise, decreased appetite, and neck pain. Owing to concerns of SCJI recurrence, I&D and intramedullary drilling were done. Notable purulence was not appreciated, decreasing concerns for reinfection. She was placed on IV vancomycin and then oral clindamycin for 6 weeks.

Throughout follow-up, the patient had tenderness and limited range of motion of the shoulder. Repeat MRI, 3 months after initial I&D, showed medial clavicular osteomyelitis (Figure 3C). This prompted a third I&D with the use of tobramycin-impregnated calcium sulfate beads. The patient was restarted on oral clindamycin for 6 weeks. She had improved range of motion and no signs of recurrence at her most recent follow-up.

### Patient 3

Patient 3 is a 14-year-old adolescent boy who presented to the emergency department (ED) with right shoulder pain and fever for three days. The patient was COVID-19–negative. Examination revealed tenderness over the right clavicle and pain with shoulder abduction. Radiographs were normal, and the patient was discharged with a suspected viral illness.

**Figure 4:**
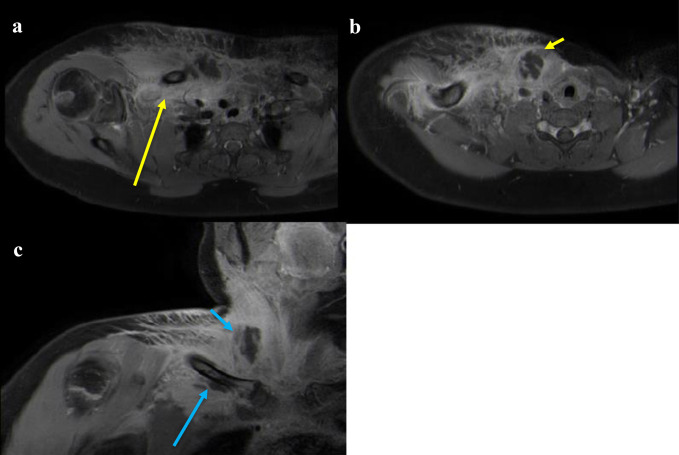
Patient 3 preoperative MR imaging. **A,** T1 postcontrast axial image demonstrating findings of right-sided SCJI including subperiosteal fluid collection and associated chest wall edema with possible mediastinitis (long arrow). **B,** T1 postcontrast axial image demonstrating spread of infection to adjacent structures with right sternocleidomastoid abscess (short arrow). **C,** T1 postcontrast coronal image demonstrating nonenhancing subperiosteal and sternocleidomastoid abscesses (long and short blue arrows, respectively). SCJI = sternoclavicular joint infection

Four days later, the patient presented to the orthopaedic clinic because of worsening malaise, fever, and shoulder pain. Physical examination demonstrated mild erythema and edema overlying the clavicle and extending to the sternum and base of the neck. There was tenderness to palpation at the clavicle, SCJ, base of the neck, and shoulder. There was limited range of motion because of pain with shoulder flexion and abduction; however, the patient was able to internally and externally rotate the shoulder without pain. The patient was subsequently transferred to the ED because of concerns for possible osteomyelitis and/or SCJI. He was febrile with leukocytosis (white blood cell—21) and elevated inflammatory markers (ESR—86, CRP—33). The platelet count was 304,000 (Table 1). MRI demonstrated extensive cellulitis, pyomyositis of the right sternocleidomastoid and pectoralis major, and osteomyelitis of the clavicle (Figure 4, A–C).

The patient underwent I&D with the placement of vancomycin and tobramycin-impregnated methyl methacrylate beads. Marked purulence was noted intraoperatively. Intraoperative cultures grew methicillin-sensitive *Staphylococcus aureus*. Antibiotic coverage was narrowed to cefazolin.

An additional I&D and exchange of antibiotic beads were done 2 days later. Methyl methacrylate beads were replaced with calcium sulfate beads containing vancomycin and tobramycin. At the six-week follow-up, he had complete resolution of symptoms. Radiographs revealed new bone formation, no sequestrum, and no new lucencies. His last follow-up at two and a half months after presentation showed complete resolution of symptoms and no signs of recurrence.

## Discussion

SCJIs are rare and typically occur in immunocompromised patients with risk factors such as diabetes, IV drug use, distant infection, and trauma.^12^ These risk factors are infrequently seen in the pediatric population, and none were present in our patients. In this case report, we discuss three unique presentations of rare infectious pathologies.

These cases highlight the unique difficulty of diagnosing musculoskeletal infections in the pediatric population, especially during the COVID-19 pandemic. All three of our patients were admitted during the pandemic and had delayed diagnoses. On average, it took two evaluations before the diagnosis of SCJI was considered and 5.3 days from initial symptoms to the onset of appropriate treatment. Delaying diagnosis can result in seeding of the infection to adjacent structures and in extreme cases can result in death.^2^

Recent literature has stressed the importance for clinicians to maintain a broad differential to limit misdiagnoses of rare conditions in the current pandemic. One example is the concern for increased complications of Kawasaki disease secondary to delayed diagnosis after COVID-19 workup.^13^ Although COVID-19 is currently one of the leading causes of febrile presentations to the ED, other potentially life-threatening conditions should be ruled out in a timely fashion.

Patients 2 and 3 sustained severe infections. They had a total length of hospitalization of 9 and 6 days, respectively. Both patients had marked purulence intraoperatively and required multiple I&Ds. They also had long antibiotic courses of 15 and 8 weeks, respectively. MRI of patient 3 indicated possible mediastinitis. Further delay in the initiation of treatment could have resulted in life-threatening consequences.^14^

Although the presentation of subtle-onset chest pain and fevers in children should prompt a workup for respiratory infection, the presence of an enlarging mass, concentrated erythema, and accompanied tenderness around the SC joint and clavicle should clue the provider to a bacterial infection such as SCJI. Laboratory markers and cultures can further elucidate the etiology and monitor response to treatment. Radiograph is often the first imaging modality ordered in a child presenting with shoulder or chest pain, but it is of little utility early on in SCJI. CT or MRI should be obtained because of their 93% and 100% sensitivities in diagnosis, respectively.^15^

Once diagnosed, patients should promptly begin treatment with broad-spectrum parenteral antibiotics. Clindamycin or vancomycin is preferred because of its optimal MRSA coverage. Patients should be transitioned to culture-specific antibiotics based on bone marrow aspirate from the infected site or culturable specimens from I&D. Surgical intervention with I&D is indicated if there is no clinical improvement noted within 36 hours of starting medical treatment, evidence of subperiosteal abscess, or spread of infection to an adjacent joint.^15,16^ Patients should remain on antibiotics for at least 4 to 6 weeks.^4,17^ Antibiotic regimens typically commence with parenteral antibiotics, followed by oral. The switch to oral antibiotics can be made when there is symptom resolution with normalization of CRP and white blood cell count.^18^ ESR and CRP can be trended to help determine the response to treatment and duration of the antibiotic course.^19,20^ No evidence suggests a minimum duration of parenteral antibiotics, and shorter parenteral courses were not found to have markedly different outcomes than longer ones.^21,22^

Systemic antibiotics may be insufficient as a stand-alone treatment because of their inability to reach sites with limited blood flow such as infected and necrotic bone.^23^ For this reason, I&D is often done. In two of the three patients, antibiotic-impregnated beads were placed in the surgical site. Antibiotic beads have multiple roles in clearing infection. They increase local concentrations of antibiotics in the infected tissues and reduce the potential adverse effects associated with high systemic antibiotic doses.^24,25^ They also fill dead space, therefore decreasing the risk of hematoma formation, a potential nidus of infection.^26^ The only patient in our study who had recurrence was initially irrigated and débrided without antibiotic beads. Subsequent I&D with tobramycin-impregnated calcium sulfate beads resolved the infection.^27^

## Conclusion

Our case report emphasizes the importance of timely diagnosis and treatment of SCJIs. The current COVID-19 pandemic may have played a role in the late diagnosis of each of our patients. All three patients were successfully treated with antibiotics and I&D; however, on average, they underwent multiple surgical procedures and had prolonged antibiotic courses. It is important to pay close attention to the history and physical examination to prompt advanced imaging if necessary so that bacterial infections such as osteomyelitis are not missed. Missed diagnoses can result in severe infections that require more intense medical and surgical treatment.
